# Historical Changes in Aging Trajectories of Two Aspects of Self-Perceptions of Aging

**DOI:** 10.1093/geroni/igaa057.2028

**Published:** 2020-12-16

**Authors:** Oliver Huxhold, Svenja Spuling, Susanne Wurm

**Affiliations:** 1 German Centre of Gerontology, Berlin, Berlin, Germany; 2 University Medicine Greifswald, Greifswald, Mecklenburg-Vorpommern, Germany

## Abstract

In recent years many studies have shown that adults with more positive self-perceptions of aging (SPA) increase their likelihood of aging healthily. Other studies have documented historical changes in individual resources and contextual conditions associated with aging. We explored how these historical changes are reflected in birth-cohort differences in aging trajectories of two aspects of SPA – viewing aging as ongoing development or as increasing physical losses. Using large-scale cohort-sequential data assessed across 21 years (N ≈ 19,000), the analyses modeled birth-cohort differences in aging trajectories of SPA from 40 to 85 years of age. The results illustrated differential birth-cohort differences: Later-born cohorts may experience more potential for ongoing development with advancing age than earlier-born cohorts. However, later-born cohorts seem to view their own aging as more negative than earlier-born cohorts during their early forties but may associate their aging less with physical losses after the age of fifty.

